# Peroxisome Proliferator Activated Receptor Gamma Sensitizes Non-small Cell Lung Carcinoma to Gamma Irradiation Induced Apoptosis

**DOI:** 10.3389/fgene.2019.00554

**Published:** 2019-06-13

**Authors:** Simran Kaur, Alo Nag, Gurudutta Gangenahalli, Kulbhushan Sharma

**Affiliations:** ^1^Division of Stem Cell and Gene Therapy Research, Institute of Nuclear Medicine and Allied Sciences, New Delhi, India; ^2^Department of Biochemistry, University of Delhi, New Delhi, India

**Keywords:** PPARG, radiosensitization, NSCLC, BAX, TP53, Hedgehog signaling

## Abstract

The nuclear receptors known as peroxisome proliferator activated receptor gamma (PPARG) are lipid-activated transcription factors that have emerged as key regulators of inflammation. PPARG ligands have been shown to have an anti-proliferative effect on a variety of cancers. These ligands can induce apoptosis via TP53 (Tumor protein p53) or ERK1/2 (Extracellular signal-regulated kinases 1/2) (EPHB2) pathways. However, the exact mechanism is not known. PPAR, a type II nuclear hormone receptor deserves attention as a selective target for radiotherapy. Our study examines the potential of selective agonism of PPARG for radiation therapy in non-small cell lung carcinoma (NSCLC). We found that the overexpression of PPARG protein as well as its induction using the agonist, rosiglitazone was able to stimulate radiation-induced cell death in otherwise radio resistant NSCLC A549 cell line. This cell death was apoptotic and was found to be BAX (BCL2 associated X) mediated. The treatment also inhibited radiation-induced AKT (Protein Kinase B) phosphorylation. Interestingly, the ionising radiation (IR) induced apoptosis was found to be inversely related to TP53 levels. A relatively significant increase in the levels of radiation induced apoptosis was observed in H1299 cells (TP53 null) under PPARG overexpression condition further supporting the inverse relationship between apoptosis and TP53 levels. The combination of PPARG agonist and radiation was able to induce apoptosis at a radiation dose at which A549 and H1299 are radioresistant, thus confirming the potential of the combinatorial strategy. Taken together, PPARG agonism was found to invigorate the radiosensitising effect and hence its use in combination with radiotherapy is expected to enhance sensitivity in otherwise resistant cancer types.

## Introduction

Non-small cell lung carcinoma (NSCLC) is one of the serious causes of death worldwide which accounts for 80% of all lung cancer in men and women with more chances in less developed countries (North and Christiani, 2013). There is an increased risk of NSCLC (specifically adenocarcinoma) in women than in men (Blot and McLaughlin, 2004). Chemotherapy can prolong the survival of such patients, but it is associated with adverse side effects (Pearce et al., 2017). Epidermal growth factor receptor (EGFR) tyrosine kinase has been found to be overexpressed in NSCLC (Prabhakar, 2015). Gefitinib, an epidermal growth factor receptor (EGFR) inhibitor has been shown to reduce tumor size in NSCLC that express high levels of EGFR receptor (Yang et al., 2017). However, these drugs can only be administered specifically to patients with EGFR overexpression (Nurwidya et al., 2016). Therefore, the development of molecules that can target NSCLC, independent of EGFR mutation is essential.

PPARs (Peroxisome proliferator activated receptors) belong to the nuclear hormone receptor family (Clarke et al., 1999). The name of PPAR has been derived from the fact that they are activated by peroxisome proliferators (Monsalve et al., 2013). PPARs have been classified into three isoforms, alpha, beta, and gamma. All of these forms show differential tissue expression (Brun et al., 1996). PPARG has a prominent role to play in adipogenesis and is further classified into two isoforms, gamma 1 (G1) and gamma 2 (G2). PPARG2 has additional 30 amino acids at its N terminal as compared to PPARG1 isoform (Tontonoz and Spiegelman, 2008). This distinct form of PPARG arises due to the alternative promoter usage and tissue-specific differences in splicing pattern (Chen et al., 2006).

PPARs are ligand activated transcriptional factors. Upon ligand binding, PPAR heterodimerizes with RXR, and the complex translocates into the nucleus (Bardot et al., 1993). The complex further binds to other cofactors to enhance the transcription of target genes. PPARs also act as lipid sensors and play a crucial role in lipid metabolism (Berger and Moller, 2002). PPARG plays a predominant role in adipogenesis and maintains the sensitivity to insulin. Due to this, TZD class of drugs that act as PPARG agonist has been widely used as insulin-sensitizing drugs in type-2 diabetes (Chiarelli and Di Marzio, 2008). PPAR can have a diverse role in physiology as evident from the fact that different isoforms have differential tissue expression and elicit different responses (Janani and Ranjitha Kumari, 2015).

PPARG ligands have been shown to have anti-proliferative effect in a variety of cancers including breast, colon, prostate, bladder, lung and ovarian cancer (Li et al., 2006; Annicotte et al., 2007; Mansure et al., 2009; Dai and Wang, 2010; Kotta-Loizou et al., 2012; Luo et al., 2015). PPARG ligands, including troglitazone and pioglitazone, have been shown to have anti-tumor effect when administered in combination with paclitaxel and cisplatin in NSCLC. Paclitaxel and cisplatin themselves lead to enhanced expression of PPARG, and the administration of PPARG ligands in combination has been shown to be more effective (Reddy et al., 2008). PPARG can exhibit anti-tumor effects both by PPARG dependent and independent pathways (Han and Roman, 2006; Cheon et al., 2009; Bolden et al., 2012). Drugs like γ tocopherol have shown to upregulate PPARG level in SW 480 colon cancer cell line attributing to the anti-cancer effect which is dependent on PPARG (Campbell et al., 2003). Prostaglandin of the J series including 15deoxy ^12,14^PGJ_2_ (PPARG ligand) induces apoptosis in breast cancer cell line by binding to PPRE response (through binding to PPARG) which suggests that peroxisomal proliferator receptor element (PPRE) mediated signaling is essentially required to initiate apoptosis in cancerous cells (Clay et al., 2001). Decreased expression of PPARG and increased expression of PTGS2 (Prostaglandin Endoperoxide Synthase 2) has also been shown to be associated with the prognosis of lung cancer, treatment with PPARG agonists lead to a decrease in the levels of PTGS2 thus inhibiting the growth of NSCLC (Hazra et al., 2008).

The potential of PPARG in combination with radiation, against cancer cell has not been investigated in detail. In this study, we evaluated the effect of PPARG in radiosensitisation of NSCLC line. Interestingly, our findings revealed that PPARG overexpression as well as the treatment with its agonist, rosiglitazone had radio sensitizing effect in otherwise radio resistant A549 cell line. We also demonstrated that PPARG mediated apoptosis in NSCLC is inversely related to TP53. Our results highlight the importance of PPARG agonists in improving radiotherapy strategies to treat lung cancer.

## Materials and Methods

### Cells, Antibodies and Reagents

A549 and H1299 (adenocarcinomic human alveolar basal epithelial cell line which is a NSCLC cell line) were obtained from NCCS (National Center for Cell Science). The cells were grown in high glucose Dulbecco’s modified Eagle’s medium (DMEM) supplemented with 10% fetal bovine serum (Gibco, Thermo Fischer Scientific). Anti PPARG (CST#2435), anti-BAX (CST#2772), anti-BCL2 (CST#2872) and anti-CASP3 (CST#9665), anti CDKN1A (CST#2947), phospho AKT (CST#9271), AKT (CST#9272), anti-CDKN1B (CST#3698), anti TP53 (CST#48818), ACTB (CST#4970), phospho P44/42 (CST#9101), and P44/42 (CST#4695) antibodies were purchased from cell signaling technologies. PARP1 (MA5-15031) was purchased from pierce. Anti rabbit FITC (#65-6111) was procured from Thermo Fischer Scientific. GAPDH (sc-25778) was obtained from Santa Cruz. Gamma H2AX antibody (MABE205) was purchased from Merck. Rosiglitazone (CAS number-122320-73-4), PI (CAS number-25535-16-4) and Annexin V-PI kit (APOAF-50TST), crystal violet (C0775) were procured from Sigma.

### Plasmid

pcDNA flag PPARG (7042 bp) was obtained from Addgene with vector backbone pcDNA3.1 myc -His A (plasmid number-8895).

### Radiation Treatment

Cells were γ irradiated using Bhabhatron cobalt-60 Teletherapy machine (Panacea Medical Technologies, Bangalore, India). Cells were irradiated 24 h post-transfection with PPARG construct at a radiation dose of 5 Gy and assessed for various parameters 24 h post-irradiation, i.e., 48 h after transfection.

### Transient Transfection

Approx. 5 × 10^5^ cells were plated on 60 mm plates. Transient transfections were performed with 5 μg of expression vector using the LipofectAMINE Reagent (Life Technologies, Inc). After 4 h incubation, the OptiMEM media was replaced with the complete media and the cells were grown for 48 h after transfection. The transfection was also performed in other formats, e.g., 6 well plates and 96 well plates by varying the cell number and amount of plasmid DNA according to the surface area.

### Analysis of Apoptosis

FACS was primarily used to study apoptosis. Cells were stained with fluorescein isothiocyanate conjugated Annexin V fluorescent labeled and PI dye. Cells negative for both Annexin V and PI were considered as live cells, Annexin V positive but PI negative represented early apoptotic cells whereas both Annexin V and PI positive cells were counted to be in the later stages of apoptosis. To determine cell death, only PI uptake was assessed by adding 2 μg/ml of PI to the cell pellet which was incubated for 10 min and analyzed by flow cytometry.

### Cell Cycle Analysis

Cells were rinsed with Dulbecco’s phosphate buffer saline (HIMEDIA), trypsinized and resuspended in HGD with 10% fetal bovine serum, collected by centrifugation, washed with Dulbecco’s phosphate buffer saline, again collected by centrifugation, resuspended in 70% ethanol and fixed at -20 degree Celsius overnight. Cells were pelleted again by centrifugation resuspended in 200 μg/ml of RNase solution, incubated at 37 degree Celsius for 45 min, added 50 μg/ml of PI solution to the incubated samples, followed by another incubation of 10 min in the dark. Flow cytometry was performed on LSR (BD Biosciences).

### Western Blot Analysis

Cell protein extraction and Western blot analysis were done using standard procedures. Breifly, protein samples were mixed with 1x lamelli buffer (Tris–HCl pH 6.8, 10% glycerol, 2% SDS, 5% beta mercaptoethanol, 0.1% bromophenol blue), boiled at 100°C for 5 min and loaded onto a SDS polyacrylamide gel in electrophoresis buffer containing 25 mM Tris–HCl pH 8.8, 250 mM Glycine and 0.1% SDS. Proteins were transferred onto a PVDF membrane. The membranes were immunoblotted with specific primary antibodies overnight at 4°C, following incubation with secondary antibody (horseradish peroxidase conjugated anti-rabbit or anti-mouse IgG, 1:3000 dilution, Santa Cruz Biotechnology), and the proteins were visualized using the chemiluminescent kit (GE).

### MTT and Cell Count Assay

For metabolic viability, cells were trypsinised; counted using hemocytometer and 2000 cells/well were seeded in each well. Transfection was performed 24 h after seeding, followed by irradiation after subsequent 24 h. Next, after 24 h post irradiation, 200 μl of 0.5 mg/ml 3-(4,5-Dimethylthiazol-2-Yl)-2,5-Diphenyltetrazolium Bromide (MTT) dissolved in PBS was added to each well for 2 h. The formazan crystals were dissolved using 200 μl of DMSO, and the absorbance at 570 nm was obtained using 96 well plate reader (BioTek, Life science instrumentation).

The cell number was assessed using hemocytometer after PPARG transfection. Approx. 2.5 × 10^5^ cells were seeded in 30 mm petri dish and the effect of treatment on cell number was determined at indicated time.

### SRB Assay

Cells were trypsinised; counted using hemocytometer and 2000 cells/well of 96 well plate were seeded in each well. Transfection was performed 24 h after seeding, followed by irradiation after subsequent 24 h. After 24 h of irradiation, 30% TCA was added to the wells and kept at 4 degree celsius for 1 h. After 1 h, 0.4% SRB (Sulforhodamine B) dissolved in 1% acetic acid was added to each well and incubated for 30 min at 37 degree celsius. Excess stain was removed by washing with 1% acetic acid followed by dissolving bound SRB with 10 mM tris salt. The absorbance was taken at 510 nm using 96 well plate reader (BioTek, Life science instrumentation).

### Clonogenic Assay

Colony formation in A549 cells was checked at a radiation dose of 2 Gy, 4 Gy, 6 Gy, 8 Gy, and 10 Gy without RSZ treatment and with RSZ treatment at a concentration of 20 μm in a 60 mm plate. The plates were kept for approx. 10–15 days for colony development followed by staining with crystal violet. The colonies so formed were counted and the survival fraction was calculated for each.

Survival fraction = Number of colonies obtained/Number of cells seeded × P.E.

### Knockdown by siRNA

Approx. 0.5 × 10^6^ cells were plated for 60 mm plate, and 5000 cells were plated in 96 well plate. For PPARG knockdown, PPARG was co-transfected along with its siRNA (10 ng) in OptiMEM media. Four hours after transfection OptiMEM media was replaced with high glucose DMEM media containing 10% FBS. The extent of knockdown was checked by Western blotting at 24 and 48 h after transfection.

### Gamma H2AX Assay

Approx. 0.25 × 10^6^ cells were grown on a cover slip. Transient transfections were performed with 5 μg of expression vector using the LipofectAMINE Reagent (Life Technologies, Inc). After 4 h incubation, the OptiMEM media was replaced with the complete media and the cells were grown for 24 h after transfection. After 24 h of transfection, radiation was given and 1 h post-irradiation cells were processed for gamma H2AX. Cells were fixed and permeabilized with 1:1 acetone: methanol solution, followed by blocking with 1% BSA and incubation with gamma H2AX primary antibody for 2 h. Cover slips were washed with PBST and incubated with secondary antibody tagged with FITC for 1 h followed by washing with PBST. Cover slips were mounted by anti-fade reagent containing DAPI on glass slide and were visualized under florescent microscope (Metafer microscope).

### Statistical Analysis

Data are expressed as means ± SD and were analyzed by analysis of variance (ANOVA) or unpaired Student’s *t*-test, as appropriate. ANOVA-Bonferroni *post hoc* tests were applied to assess significant differences between groups. A *p*-value ≤ 0.05 was considered statistically significant.

## Results

### Overexpression of PPARG Induces Cell Death in Human Non-small Cell Lung Carcinoma (NSCLC)

**FIGURE 1:**
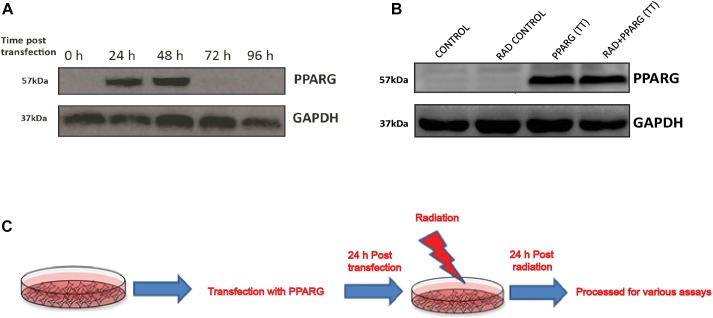
Western blot analysis for the overexpression of PPARG in A549 cell line. **(A)** Cell lysates from different time point post PPARG transfection were run on SDS PAGE and probed with mAb for PPARG. The blots were developed using chemiluminescence. **(B)** Overexpression of PPARG (TT-transient transfection) was evaluated in irradiated (5 Gy) and non-irradiated conditions in A549 cells. **(C)** Detailed plan used for experiments to assess the effect of PPARG overexpression on A549 cell line in combination with radiation.

A549 cells were transiently transfected with PPARG gene expression plasmid (Addgene# 8895). The optimum expression of PPARG was seen 24 h post-transfection and was maintained till 48 h (Figure 1A). Cells were irradiated 24 h post-transfection, and the response was determined 24 h post-irradiation (48 h post PPARG transfection). The endogenous expression of PPARG was negligible in A549 cells (Figure 1B). Cells over-expressing PPARG after transient transfection, either alone or combined with radiation exposure, showed comparable levels of PPARG. The scheme of experimentation has been shown in Figure 1C.

**FIGURE 2:**
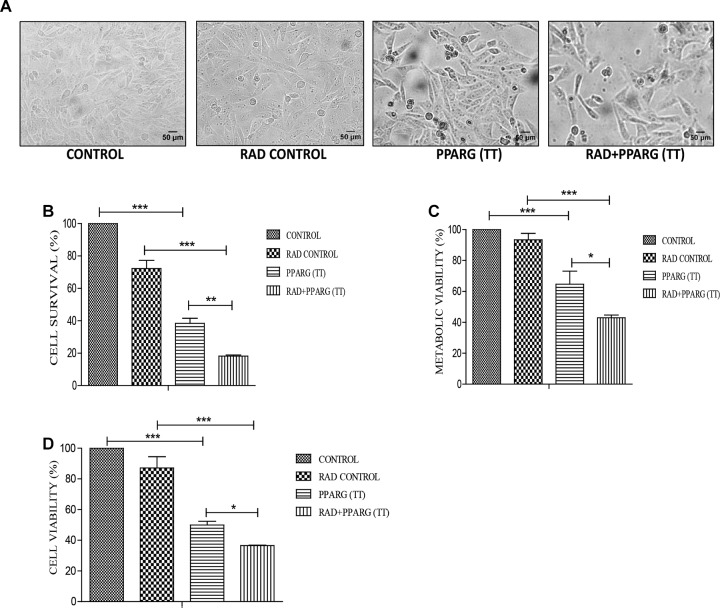
PPARG radiosensitize A549 cells. A549 cells were transfected with PPARG and irradiated 24 h post-transfection and processed 48 h after transfection. The groups included CONTROL, RAD CONTROL (5 Gy), PPARG (TT), and RAD+PPARG (TT). The cells were processed for various assays. **(A)** Morphological analysis was done 24 h post-radiation (48 h post PPARG transfection). **(B)** Cell count was determined using hemocytometer 24 h post-radiation. The error bar represents standard deviation in which ^∗^*P* < 0.05, ^∗∗^*P* < 0.01, and ^∗∗∗^*P* < 0.001. The experiment was done in triplicate (*n* = 3) and repeated three times **(C)** cell viability was assessed by MTT assay 24 h post-radiation. The error bar represents standard deviation in which ^∗^*P* < 0.05, ^∗∗^*P* < 0.01, and ^∗∗∗^*P* < 0.001. The experiment was done in triplicate (*n* = 3) and repeated three times. **(D)** Cell viability was assessed by SRB (Sulforhodamine) assay 24 h post-radiation. The error bar represents standard deviation in which ^∗^*P* < 0.05, ^∗∗^*P* < 0.01, and ^∗∗∗^*P* < 0.001. The experiment was done in triplicate (*n* = 3).

Cells were relatively more stressed morphologically in both PPARG as well as PPARG+radiation condition as compared to radiation alone (Figure 2A). Cell survival, as well as metabolic viability, was reduced 48 h post PPARG-transfection. The percentage inhibition in cell survival was 25% in radiation alone which on PPARG transfection increased to 62%, it was further increased to 82% when PPARG was combined with radiation (Figure 2B). The percentage reduction in cell viability was 7% in radiation alone which increased to 35% in PPARG treatment; it further increased to 57.1% in radiation+PPARG group (Figure 2C). Additionally, the percentage inhibition as determined by SRB assay was found to be 13% in radiation alone which on PPARG transfection increased to 50%; it further increased to 65% when PPARG was combined with radiation (Figure 2D). The noticeable difference in percentage values among the two assays may be attributed to the variable mitochondrial activity and biogenesis. The combination of PPARG and radiation led to further reduction in metabolic viability as well as survival (Figure 2B,C). These results demonstrated that both metabolic viability, as well as cell survival, is reduced upon PPARG treatment, and the combination of radiation with PPARG leads to a further reduction in these parameters. This part of our study suggested that the combination of PPARG with radiation can enhance radiosensitisation in NSCLCs.

### Radiosensitization Induced by the Combinatorial Treatment of PPARG and Radiation Is BAX Mediated

**FIGURE 3:**
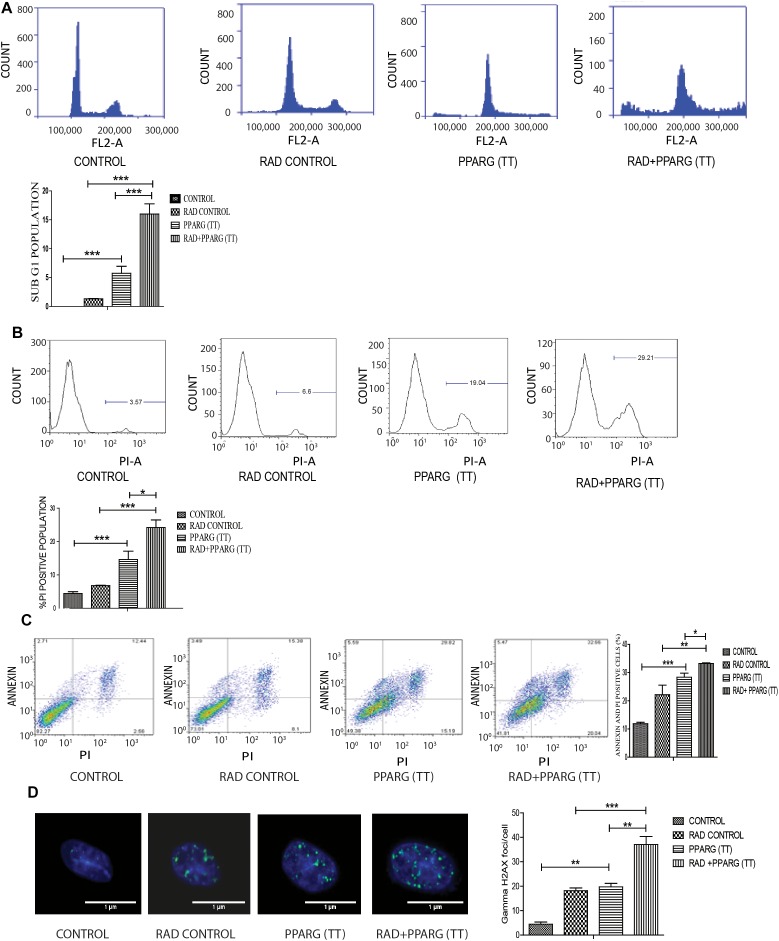
Cell death induced by PPARG is apoptotic-c in nature. A549 cells were transfected with PPARG (PPARG (TT) and irradiated 24 h post-transfection and processed 48 h after transfection. These samples which includes CONTROL, RAD CONTROL (5 Gy), PPARG (TT), and RAD+PPARG (TT) were processed for various assays. **(A)** Sub G1 phase analysis was performed using flow cytometry, 48 h post-transfection. The plot on the right shows quantification of the same. **(B)** PI uptake was performed 48 h post-transfection, and the quantification has been shown on the right. The error bar represents standard deviation in which ^∗^*P* < 0.05, ^∗∗^*P* < 0.01, and ^∗∗∗^*P* < 0.001. The experiment was done in triplicate (*n* = 3) and repeated three times. **(C)** Annexin PI (Propidium iodide) was performed to evaluate apoptosis, 48 h post-transfection. FL1-H represents the filter for FITC whereas FL2-H represents filter for PI. The quantitation has been shown in column graph. The error bar represents standard deviation in which ^∗^*P* < 0.05, ^∗∗^*P* < 0.01, and ^∗∗∗^*P* < 0.001. The experiment was done in triplicate (*n* = 3) and repeated three times **(D)** Gamma H2AX foci were measured 1 h post-irradiation in A549 cells and average foci/cell was calculated.

As demonstrated earlier, both PPARG and PPARG+radiation resulted in reduced NSCLC survival. To confirm this, sub G1 population (an indicator of cell death) was determined. Cell cycle analysis showed that there was only 1.2% increase in sub G1 population in radiation alone group of A549 cells as compared to control indicating their radio-resistant nature. On PPARG treatment, the sub G1 population went up to 5.5%, which further increased to 15.83% in the combination of PPARG with radiation clearly suggesting the potential of this combination against resistant lung cancer cells (Figure 3A). The effect of PPARG transfection on different phases of cell cycle has been shown in Supplementary Figure S1. To determine the overall effect of different experimental groups on the cell viability, their ability to take up PI (an indication of dead cells) was determined. Radiation alone led to 6.79% increase in PI positive cells, whereas PPARG and combination of PPARG with radiation led to 12.64 and 22.01% increase in PI positive population, respectively, further supporting our earlier observations (Figure 3B). Vector alone did not have much effect on PI uptake indicating that the effect is not due to lipofectamine toxicity (data not shown). DNA damage has been considered as an important effect of radiation exposure (Ward, 1988). The DNA damage was found to be prominent when radiation was combined with PPARG. It was indicated by the average gamma H2AX foci/cell which increased significantly when radiation was combined with PPARG (Figure 3D).

**FIGURE 4:**
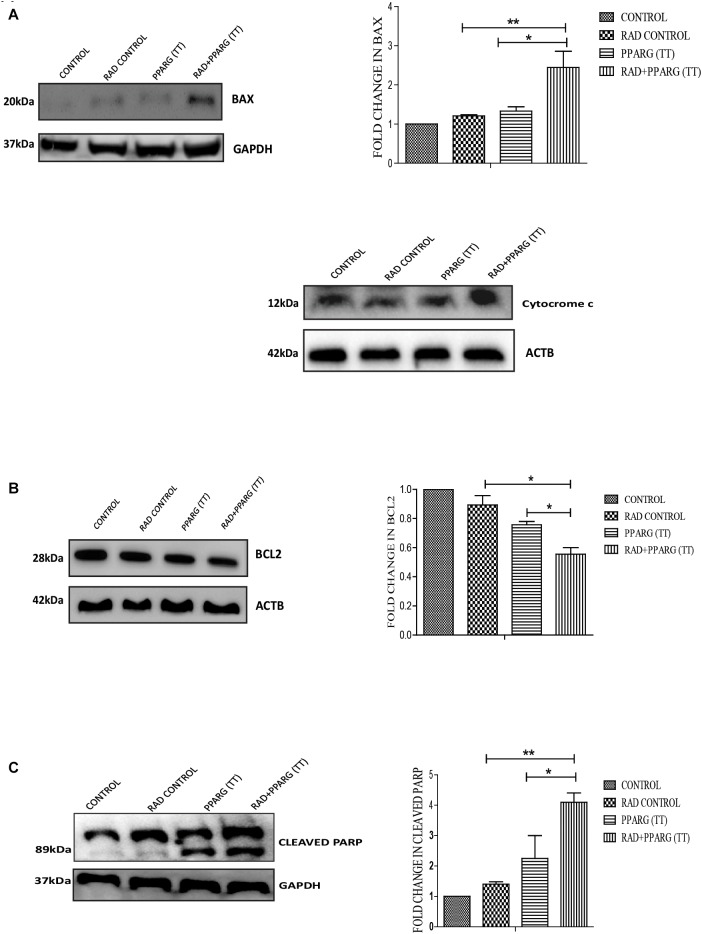
Death induced by PPARG in combination with radiation is BAX mediated. A549 cells were transfected with PPARG (PPARG (TT) and irradiated 24 h post-transfection, and processed 48 h after transfection. These samples which include CONTROL, RAD CONTROL (5 Gy), PPARG (TT), and RAD+PPARG (TT) were processed for Western blotting **(A)** The effect of PPARG overexpression on the levels of BAX and cytocrome c was evaluated using Western blotting. GAPDH was used as a loading control. The graph represents the relative quantification of BAX. The protein level has been normalized with loading control. The error bar represents standard deviation in which ^∗^*P* < 0.05, ^∗∗^*P* < 0.01, and ^∗∗∗^*P* < 0.001 **(B)** The effect of PPARG overexpression on the levels of BCL2 was evaluated using Western blotting. ACTB was used as a loading control. The graph represents the relative quantification of BCL2. The protein level has been normalized with the loading control. The error bar represents standard deviation in which ^∗^*P* < 0.05, ^∗∗^*P* < 0.01, and ^∗∗∗^*P* < 0.001. The blots were repeated three times. **(C)** The effect of PPARG overexpression on the levels of cleaved PARP, 48 h post-transfection was evaluated using Western blotting. GAPDH was used as a loading control. The graph represents the relative quantification of cleaved PARP levels where the protein level has been normalized with loading control. The error bar represents standard deviation in which ^∗^*P* < 0.05, ^∗∗^*P* < 0.01, and ^∗∗∗^*P* < 0.001. The blots were repeated three times.

To understand whether the kind of cell death observed is apoptotic, Annexin-PI assay was performed. Annexin directly indicates cells undergoing apoptosis. There was increased apoptosis in the combination of PPARG+radiation as indicated by Annexin PI staining (Figure 3C). BAX plays a major role in apoptotic induction (Pawlowski and Kraft, 2000). To evaluate the role of BAX in the apoptosis induced by the combination, we tested its levels by Western blotting. In addition to BAX, the intrinsic pathway of apoptosis is regulated by the BCL2 family of proteins. BCL2 proteins can be either proapoptotic or prosurvival (Shamas-Din et al., 2013). The critical balance between these proteins determines the fate of the cell (Kale et al., 2018). The level of pro-apoptotic protein BAX was found to be 2–3 fold higher when PPAR was combined with radiation as compared to PPARG alone (Figure 4A). BCL2, an anti-apoptotic protein was found to be slightly reduced as compared to PPARG treated samples. A noticeable difference in the levels of BCL2 was observed between PPARG alone and PPARG+radiation (Figure 4B). To verify if the death induced by PPAR and its combination with radiation was apoptotic, we tested the levels of cleaved PARP. PPARG alone induced a two-fold increase in levels of cleaved PARP whereas the four-fold increase was observed in the combination of PPARG with radiation (Figure 4C). The increase in apoptotic markers signifies that the combination of PPARG with radiation enhances the radiosensitising effect even at 5 Gy (majorly through apoptosis), the dose at which there is no significant death in A549 cells. Furthermore, these results indicate that the apoptotic death induced by the combination of PPARG and radiation is BAX mediated (Figure 4).

### Radiosensitising Effect of PPAR Is Inversely Related to the TP53 Pathway

**FIGURE 5:**
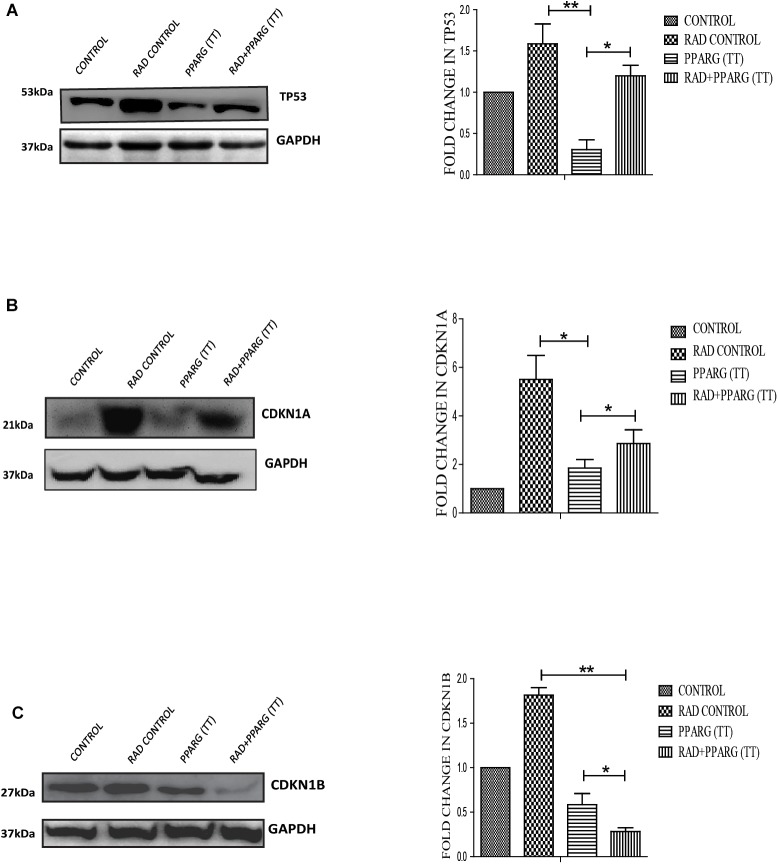
PPARG mediated apoptosis is inversely related to TP53 pathway. A549 cells were transfected with PPARG (PPARG (TT) and irradiated 24 h post-transfection, and processed 48 h after transfection. These samples which include CONTROL, RAD CONTROL (5 Gy), PPARG (TT), and RAD+PPARG (TT) were processed for Western blotting. **(A)** The effect of PPARG overexpression on the levels of TP53 was evaluated using Western blotting. Same GAPDH was used as a loading control as that for PPARG blot in Figure 1. The graph on the right represents the relative quantification of TP53. The protein level has been normalized with loading control. The error bar represents standard deviation in which ^∗^*P* < 0.05, ^∗∗^*P* < 0.01, and ^∗∗∗^*P* < 0.001. The blots were repeated three times. **(B)** The effect of PPARG overexpression on the levels of CDKN1A, the downstream target of TP53 was evaluated using Western blotting. GAPDH was used as a loading control. The graph represents the relative quantification of CDKN1A. The protein level has been normalized with loading control. The error bar represents standard deviation in which ^∗^*P* < 0.05, ^∗∗^*P* < 0.01, and ^∗∗∗^*P* < 0.001. The blots were repeated three times. **(C)**. The effect of PPARG overexpression on the levels of CDKN1B was evaluated using Western blotting. GAPDH was used as a loading control. The graph on the right represents the relative quantification of CDKN1B. The protein level has been normalized with loading control. The error bar represents standard deviation in which ^∗^*P* < 0.05, ^∗∗^*P* < 0.01, and ^∗∗∗^*P* < 0.001. The blots were repeated three times.

Since TP53 is an important modulator of apoptosis and is known to get activated in radiation induced DNA damage conditions (Verheij and Bartelink, 2000), it became important to investigate the role of TP53 in PPARG mediated apoptosis. Surprisingly, the expression of TP53 reduced significantly after PPARG transfection in A549 cells (Figure 5A). To confirm this, the levels of downstream targets of TP53 including CDKN1A and CDKN1B were explored (Figure 5). CDKN1A is the direct target of TP53 mediated transcription. TP53 translocates to the nucleus and dimerises to mediate the transcription of CDKN1A by binding to its promoter region (Ahn et al., 2007). Although TP53 also regulates CDKN1B, it may also be regulated through the PI3K pathway (Kim et al., 2009). Similar to TP53, levels of CDKN1A were found to be significantly reduced after overexpression of PPARG. In comparison to CDKN1A, the reduction in the levels of CDKN1B was relatively weaker under PPARG overexpression conditions. Interestingly, there was a profound reduction in CDKN1B under PPARG+radiation combination, which may be due to its dual regulation (Figure 5C).

### Similar to Its Overexpression, PPARG Agonist Induces Radiosensitisation in A549 Cells

**FIGURE 6:**
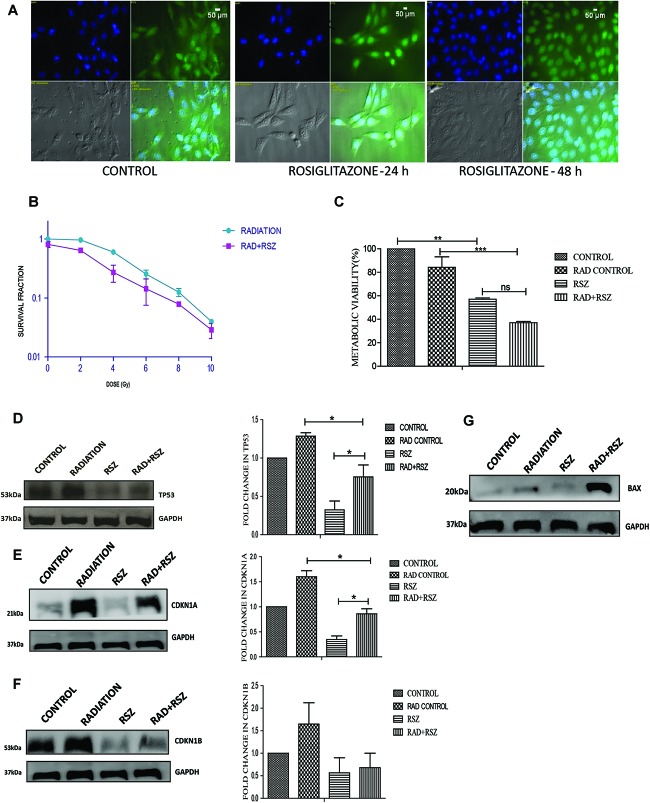
Similar to its overexpression, PPARG ligand rosiglitazone (RSZ) also induce radiosensitization. A549 cells were treated with agonist of PPARG rosiglitazone (RSZ). The samples which included CONTROL, RADIATION (5 Gy), RSZ, and RAD+RSZ were processed for various assays **(A)** PPARG translocate to nucleus partially at 24 h and fully after 48 h of treatment with rosiglitazone as indicated by immunofluorescence **(B)** SF-2 value was calculated on treatment of A549 cells at different doses of radiation along with the treatment of cells with rosiglitazone for 48 h. The experient was performed in triplicate. **(C)** viability of A549 cell line was assessed 24 h post-radiation with continuous treatment with rosiglitazone for 48 h in A549 cell line. The error bar represents standard deviation in which ^∗^*P* < 0.05, ^∗∗^*P* < 0.01, and ^∗∗∗^*P* < 0.001. **(D–F)** The effect of PPARG ligand on the levels of TP53 and its downstream target CDKN1A and CDKN1B was evaluated using Western blotting. GAPDH was used as a loading control which is same for CDKN1A and CDKN1B. The graph on the right represents the relative quantification of the blots. The protein level has been normalized with loading control. The error bar represents standard deviation in which ^∗^*P* < 0.05. The blots were repeated two times. **(G)** The effect of PPARG ligand on the levels of BAX was evaluated using Western blotting. GAPDH was used as a loading control.

Rosiglitazone is a well-known inducer of PPARG (Saramaki et al., 2006). A 20 μm concentration of rosiglitazone was sufficient to induce nuclear translocation of PPARG and was thus used for further experiments. As determined by translocation studies, rosiglitazone led to the induction of PPARG; after 48 h of treatment with rosiglitazone most of PPARG is localized in the nucleus (Figure 6A). To evaluate the effect of PPARG agonist in A549 cells, the cells were exposed to different doses of radiation in the presence of rosiglitazone. The survival fraction was drastically reduced in a radiation dose-dependent manner when a drug was given in combination. As compared to radiation alone, the relative SF-2 value came down from 1 to 0.65 in the combination (Figure 6B). The relative difference was maximum at a dose of approx. 5 Gy and thus, this dose was chosen for further experiments. As expected, the viability of cells was significantly reduced at a radiation dose of 5 Gy, and it went down from 81% in the radiation control to 68% in radiation+rosiglitazone treatment (Figure 6C). More cell death with the overexpression of PPARG as compared to rosiglitazone can be because of the more direct effect of the overexpression as compared to its analog. This indicates that PPARG agonists may induce a PPARG overexpression like radiosensitisation effect in A549 cells. Also, the molecular mechanism of sensitisation was very similar as indicated by a common trend in the levels of TP53, CDKN1A as well as CDKN1B (Figure 6D–F). Furthermore, the levels of BAX indicated that Rosiglitazone mediated cell death is also mediated through it as represented in Figure 6G.

### PPARG Overexpression Induces Enhanced Apoptosis in TP53 Null Cells

**FIGURE 7:**
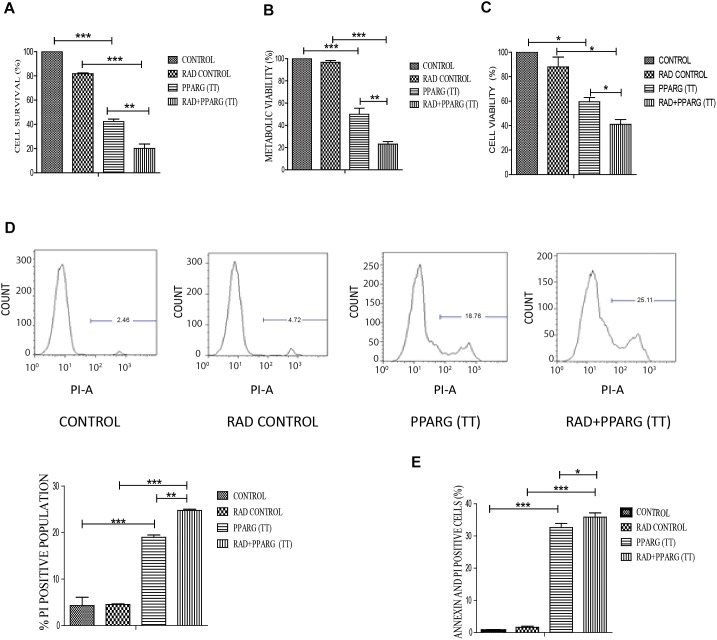
PPARG induces cell death in TP53 null cell line. H1299 cells were transfected with PPARG and irradiated 24 h post-transfection and processed 48 h after transfection. These samples which included CONTROL, RAD CONTROL (5 Gy), PPARG (TT), and RAD+PPARG (TT) were processed for various assays. **(A)** Cell survival was studied 48 h post-transfection. The error bar represents standard deviation in which ^∗^*P* < 0.05, ^∗∗^*P* < 0.01, and ^∗∗∗^*P* < 0.001. The experiment was done in triplicate (*n* = 3) and repeated three times **(B)** metabolic viability was determined by MTT 48 h post-transfection. The error bar represents standard deviation in which ^∗^*P* < 0.05, ^∗∗^*P* < 0.01, and ^∗∗∗^*P* < 0.001. The experiment was done in triplicate (*n* = 3) and repeated three times **(C)** Cell viability was determined by SRB 48 h post-transfection. The error bar represents standard deviation in which ^∗^*P* < 0.05, ^∗∗^*P* < 0.01, and ^∗∗∗^*P* < 0.001. The experiment was done in triplicate (*n* = 3). **(D)** PI uptake was performed 48 h post-transfection, and the quantification has been shown on the right. The error bar represents standard deviation in which ^∗^*P* < 0.05, ^∗∗^*P* < 0.01, and ^∗∗∗^*P* < 0.001. The experiment was done in triplicate (*n* = 3) and repeated two times **(E)** Annexin PI was performed to evaluate apoptosis, 48 h post-transfection. The quantitation has been shown in column graph. The error bar represents standard deviation in which ^∗^*P* < 0.05, ^∗∗^*P* < 0.01, and ^∗∗∗^*P* < 0.001. The experiment was done in triplicate (*n* = 3) and repeated three times.

**FIGURE 8:**
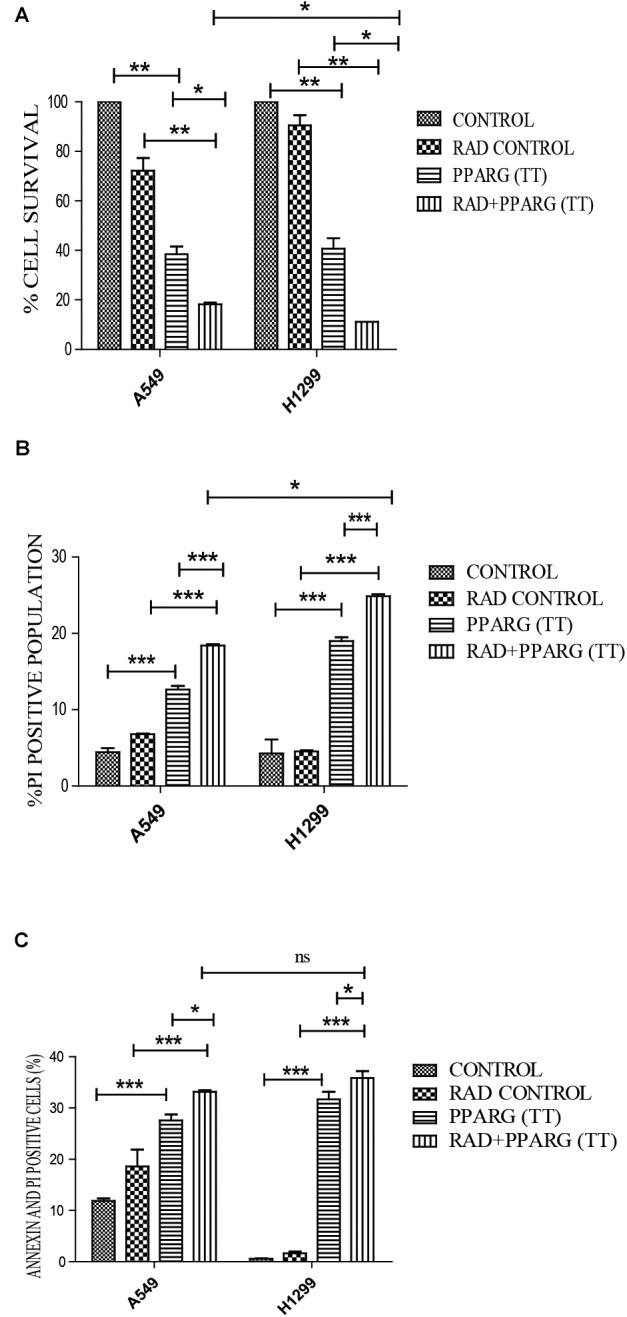
Cell death induced by PPARG+radiation is more pronounced in H1299 as compared to A549. **(A)** Comparative analysis of cell death between TP 53 null cell line and A549 was done 48 h post-transfection. The error bar represents standard deviation in which ^∗^*P* < 0.05, ^∗∗^*P* < 0.01, and ^∗∗∗^*P* < 0.001 **(B)** Comparative analysis of PI uptake was performed 48 h post-transfection. The quantitation has been shown in column graph. The error bar represents standard deviation in which ^∗^*P* < 0.05, ^∗∗^*P* < 0.01, and ^∗∗∗^*P* < 0.001 and **(C)** Comparative analysis for Annexin PI was performed to evaluate apoptosis, 48 h post-transfection. The quantitation has been shown in column graph. The error bar represents standard deviation in which ^∗^*P* < 0.05, ^∗∗^*P* < 0.01, and ^∗∗∗^*P* < 0.001.

Our earlier data indicated that PPARG overexpression, as well as its agonist, induces a reduction in TP53 levels. To confirm this, we analyzed cell survival and apoptosis in a TP53 null background using H1299 cells. These cells (devoid of TP53), showed a similar trend in cell death and apoptosis as determined by cell count, metabolic viability, SRB, PI uptake and Annexin PI (Figure 7). The cell survival decreased by 18.29% in the radiation alone whereas the reduction was 57.6% in PPARG overexpression. In the combination of PPARG+radiation, it went down by 79.87% (Figure 7A). The metabolic viability and cell viability by SRB showed a similar trend (Figure 7B,C, respectively). This reduction in cell survival in TP53 null cells after PPARG overexpression was also majorly contributed by apoptosis as indicated by PI and Annexin PI (Figure 7D,E). To confirm that PPARG induced cell death (either alone or in combination with radiation) is inversely related to TP53, we compared the relative levels of cell survival and apoptosis between A549 and H1299 (TP53 expression and TP53 null cell line, respectively). Our data clearly indicated a relative increase in the cell death as well as apoptosis after overexpression of PPARG in TP53 null cell line (Figure 8A–C), which further supports that PPARG mediated cell death is inversely related to the levels of TP53.

### Apoptosis Is Inhibited Under PPARG Knock-Down Condition

**FIGURE 9:**
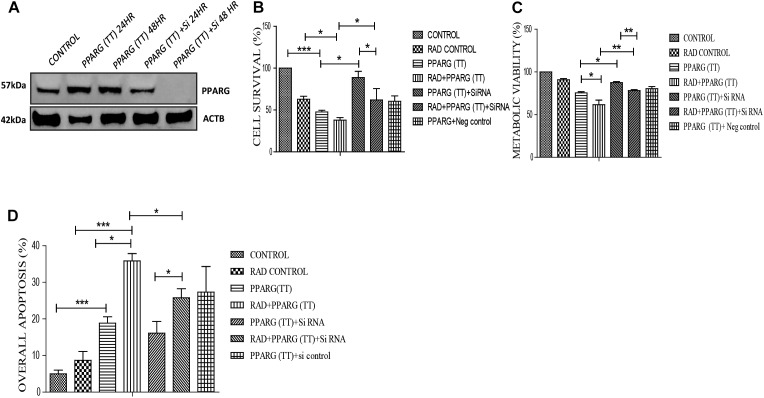
Apoptosis induction in A549 cell line is PPARG dependent. **(A)** Knockdown of PPARG was done using siRNA specific to PPARG. Effect of PPARG knockdown on A549 cells was determined by various assays. PPARG was co-transfected along with PPARG siRNA and the complete knockdown of PPARG was evaluated using Western blotting. The protein level has been normalized with loading control. **(B)** Cell survival was studied 48 h post-transfection The error bar represents standard deviation in which ^∗^*P* < 0.05, ^∗∗^*P* < 0.01, and ^∗∗∗^*P* < 0.001. The experiment was done in triplicate (*n* = 3) two times **(C)** Metabolic viability was determined 48 h post-transfection using MTT assay. The error bar represents standard deviation in which ^∗^*P* < 0.05, ^∗∗^*P* < 0.01, and ^∗∗∗^*P* < 0.001. The experiment was done in triplicate (*n* = 3) two times **(D)** Annexin PI was performed to evaluate apoptosis, 48 h post-transfection. The quantitation has been shown in column graph. The error bar represents standard deviation in which ^∗^*P* < 0.05, ^∗∗^*P* < 0.01, and ^∗∗∗^*P* < 0.001. The experiment was done in triplicate (*n* = 3).

To validate that the apoptosis was induced in PPARG dependent manner in NSCLC, we silenced PPARG using its siRNA. Maximum inhibition was observed 48 h post-transfection (Figure 9A). There was a significant reduction in PPARG mediated cell death in the presence of its siRNA confirming its direct contribution. Although not significant, a similar trend was observed in the combinatorial treatment of radiation and PPARG (Figure 9B). A very significant improvement was observed for the metabolic viability of the cells upon PPARG depletion in PPARG+radiation group (Figure 9C). The Annexin PI assay also showed that the overall apoptosis was improved significantly when PPARG was knocked down indicating that the apoptosis gets induced in a PPARG dependent manner (Figure 9D). There was a significant reduction in apoptosis in PPARG+radiation combination under PPARG knockdown conditions indicating that the apoptosis induced is PPARG mediated.

### PPARG Inhibits AKT and MAPK Pathway in NSCLC

**FIGURE 10:**
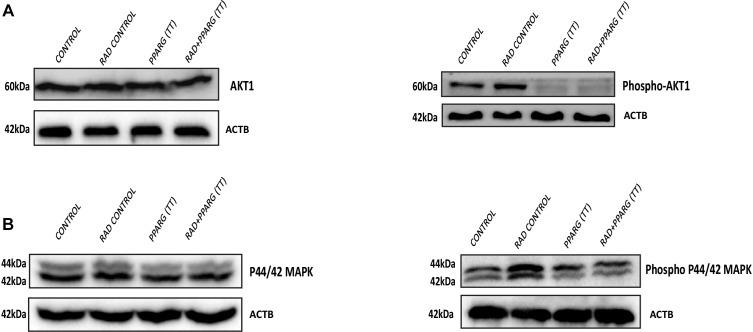
Inhibition of the survival pathways by PPARG in A549 cells. A549 cells were transfected with PPARG and irradiated 24 h post-transfection, and processed 48 h after transfection. **(A)** The effect of PPARG overexpression on the levels of total AKT and phospho AKT, 48 h post-transfection was evaluated using Western blotting. ACTB (beta actin) was used as a loading control. **(B)** The effect of PPARG overexpression on the levels of total P44/42 (MAPK) and phospho P44/42 (MAPK), 48 h post-transfection was evaluated using Western blotting. ACTB (beta actin) was used as a loading control.

AKT is considered a cell survival pathway in general (Ward, 1988). To understand the effect of PPARG and its combination with radiation on the AKT pathway, we studied the levels of AKT phosphorylation (Ser 473). We observed a very significant reduction in the levels of AKT phosphorylation thereby indicating the down regulation of this pathway during PPARG and PPARG+radiation induced cell death (Figure 10A). In addition to AKT, MAPK pathway has also been shown to be involved in cell proliferation (Shapiro, 2002). The MAPK protein phospho P44/42 (ERK1/2) controls cell proliferation leading to tumorigenic effect (Hwang et al., 2016). To study the effect of PPARG transfection on proliferation capacity of A549 cells, we explored the level of phospho P44/42 MAPK, and found it to decrease on PPARG transfection (Figure 10B). To summarize, in order to survive under radiation conditions, NSCLC may activate the pro-survival pathways e.g., AKT (Song et al., 2005) and MAPK pathway. However, the treatment of PPARG leads to the downregulation of both of these pathways further leading to the induction of apoptosis in these cells.

## Discussion

**FIGURE 11:**
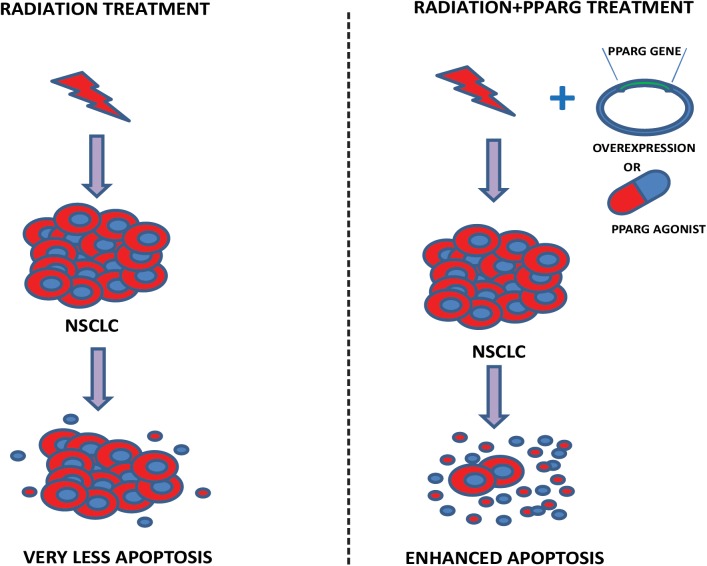
Proposed model. The model illustrates our study. In the left panel, the effect of radiation on A549 cells has been shown. As observed in our data, a very less cell death is represented. Right panel shows that the combination of either PPARG agonist or its overexpression, with radiation induces significant amount of radiosensitization leading to enhanced apoptosis in NSCLC.

In this study, we have demonstrated that A549 cells express PPARG at low levels; the overexpression of PPARG and treatment with PPARG agonist reduces their proliferative capacity. Furthermore, A549 is a radioresistant cell line; overexpression of PPARG makes this cell line more sensitive to radiation at a dose which doesn’t have any profound effect otherwise. Also, PPARG mediated apoptosis was found to be inversely related to TP53 levels in our study. Together, these results suggest that PPARG can sensitize otherwise resistant NSCLC to radiation and hence can be considered as a potential target to improve the radiotherapeutic procedure for these cancer types. A model representing the outcome of our study has been shown in Figure 11.

Radioresistance is the main cause of ineffective radiotherapy against NSCLC. Improved radiotherapeutic strategies against these resistant cancer types are the need of the hour. In our study, we explored the potential of PPARG to improve radiotherapeutic efficiency against A549, NSCLC, a known radioresistant cancer cell type. A549 can remain attached and survive even at 5 Gy radiation dose (Gomez-Casal et al., 2013). Just 25% reduction in survival as compared to other tumor cell line like MDAMB-231 which show approx. 50% survival at a radiation dose of 5 Gy (data not shown) classifies A549 cell line as a radio resistant cell line (Schaue and McBride, 2005). PPARG when combined with radiation, induced apoptosis in A549 cells at 5 Gy which was not able to otherwise induce any cell death. Our study is supported by the fact that PPARG silencing can block apoptotic induction in A549 cells (Yang, 2013). Studies have also suggested PPARG independent apoptosis in A549 cell line in the combined treatment of PPARG ligands with gamma radiation (Han et al., 2013). However, our knockdown data clearly suggested that the apoptosis induced by radiation+PPARG-overexpression is indeed PPARG dependent in A549 cell line (Figure 9).

One of the key survival pathways in radiation stress is the PI3K/AKT pathway. AKT pathway blocks the apoptotic process and keeps the cell alive in adverse microenvironment including radiation. This pathway has been shown to be activated in NSCLC (Chautard et al., 2010). We found that the overexpression of PPARG alone or its combination with radiation led to the down regulation of phospho-AKT indicating improved radiosensitisation of these cells. Additionally, MAPK pathway which has role in cell proliferation was downregulated with PPARG transfection. This data supports the fact that PPARG may inhibit survival as well as proliferation pathways leading to the induction of cell death.

We have shown PPARG and PPARG+radiation induced apoptosis in A549 cells through various markers including cleaved PARP and BAX. Ionizing radiation may induce apoptosis via TP53 activation, although there are reports of TP53 independent mechanisms as well (Scrima et al., 2012). In our study, apoptotic induction was found to be inversely related to TP53 levels. BCL2 and BAX play an important role in regulating apoptosis through mitochondrial membrane permeability and the cytochrome c release. BCL2 protein is located in the nuclear, endoplasmic reticulum and mitochondrial membrane and is anti-apoptotic whereas BAX is located in the cytoplasm and is considered as pro-apoptotic (Tsujimoto, 1998). Increased levels of BAX and decreased levels of BCL2 were observed on PPARG overexpression. Previous studies have shown that apoptosis induced by rosiglitazone is mediated through BAX (Liu et al., 2010). Another study showing rosiglitazone mediated apoptosis in NSCLC has indicated the involvement of BAX pathway (Kim et al., 2007). Our study also indicates that PPARG treatment may lead to the induction of DNA damage, as the gamma H2AX foci increased significantly in PPARG+radiation group 1 h post-radiation as compared to the radiation alone. This may also suggest that NSCLC, when combined with PPARG+radiation are not able to repair the damage leading to cell death. TP53 is a tumor suppressor protein which transcriptionally activates CDKN1A by binding to its promoter region (Abukhdeir and Park, 2008). CDKN1B is also regulated by TP53 as there are TP53 binding sites in the CDKN1B promoter region. However, reports are suggesting that CDKN1B may also be regulated by other proteins like AKT, SGK, and RSK, which promote CDKN1B sequestration in cytoplasm promoting cell proliferation and cell motility (Wander et al., 2011). Both CDKN1A and CDKN1B inhibit cyclin/CDK complexes mediating G1/S arrest (Besson et al., 2008). Earlier studies with PPARG agonists have shown increased levels of CDKN1A and CDKN1B, thereby leading to apoptosis through G1-S arrest (Liu et al., 2010). In our study, we found a decline in the levels of CDKN1A and CDKN1B which are downstream targets of TP53. The reduction in the levels of CDKN1B was less profound as compared to CDKN1A. However, when combined with radiation, the levels of CDKN1A got induced again in contrast to CDKN1B, which went down further.

It has been shown that NSCLC expresses high levels of CDKN1B, which provide them with the ability to survive under culture conditions unfavorable for cell growth such as a lack of nutrients and hypoxia (Masuda et al., 2001). In addition to TP53, CDKN1B levels may be regulated by many other pathways including PI3K, MEK etc. Further reduction in the levels of CDKN1B after treatment with PPARG+radiation may explain the enhanced radiosensitisation of these cells to radiation induced cell death.

In addition to the TP53 pathway, ionizing radiations may induce apoptosis through the activation of JNK pathways (Dent et al., 2003). JNK phosphorylates the BH-3 family proteins like BIM which may induce JNK mediated BAX/BAK apoptotic pathway (Lei and Davis, 2003). Similarly, PPAR ligands have also been shown to induce MAPK which includes JNK (Gardner et al., 2005). JNK can phosphorylate PPARG and increases its transcriptional activity (Yin et al., 2006). Also, PPARG induces PTEN expression that is involved in cell cycle arrest and apoptosis (Farrow and Evers, 2003). In our study, we have found low phospho AKT which is a direct target of PTEN. Therefore, the possibility of the involvement of these pathways in PPARG+radiation induced apoptosis in NSCLC cannot be ignored.

Hedgehog signaling has been shown to be associated with PPARG (Skoda et al., 2018). It plays a crucial role in embryonic development and may also get activated in variety of cancers including NSCLC (Abe and Tanaka, 2016; Skoda et al., 2018). The activation of Hedgehog signaling leads to the proliferation of cells through MAPK signaling pathway (Liu et al., 2018). There are studies suggesting that sonic Hedgehog signaling (SHH) leads to the phosphorylation of PPARG at ser112 and thereby lead to its degradation (Yao et al., 2019). Due to this, inhibitors of SHH signaling including Vismodegib have been used along with PPARG agonist rosiglitazone in patient samples for treating solid tumors (LoRusso et al., 2013).

The expression of PPARG is differential in normal versus cancer cells. Decreased levels of PPARG gene have been observed in patients with esophageal cancer that is correlated with poor prognosis in patients (Terashita et al., 2002). Also a study on human NSCLC has suggested that the patients expressing PPARG have better survival than those who don’t express it (Giaginis et al., 2012). Also, PPARG agonist can cross blood brain barrier and has been proven beneficial in reducing tumor in human xenograft model (Grommes et al., 2013). The role of PPARG in radiation induced apoptosis in NSCLC shown in this study can be particularly relevant in radiotherapy where resistance to radiation is the major complication. Thus, our study suggests that PPARG overexpression, as well as treatment with its agonist, can be potentially explored to radiosensitise certain otherwise resistant cancer types, e.g., NSCLC.

## Data Availability

No datasets were generated or analyzed for this study.

## Author Contributions

SK performed all the experiments. AN involved in the designing experiments. GG helped in designing experiments. KS planned the study and helped in performing the experiments.

## Conflict of Interest Statement

The authors declare that the research was conducted in the absence of any commercial or financial relationships that could be construed as a potential conflict of interest.

## Supplementary Material

FIGURE S1 PPARG treatment led to accumulation of cells in S phase which on treatment with PPARG+radiation move toward cell death. Various phases of cell cycle after different treatment has been depicted. A549 cells were transfected with PPARG (PPARG (TT) and irradiated 24 h post-transfection, which is 48 h after transfection. These samples which included CONTROL, RAD CONTROL (5 Gy), PPARG (TT), and RAD+PPARG (TT) were processed for cell cycle analysis. The error bar represents standard deviation in which ^∗^*P* < 0.05, ^∗∗^*P* < 0.01, and ^∗∗∗^*P* < 0.001. The experiment was done in triplicate (*n* = 3).

## Abbreviations

AKT: protein kinase B
BAX: BCL2 associated X, apoptosis regulator
BCL2: B-cell lymphoma 2, apoptosis regulator
C4-2: human papillomavirus-related cervical squamous cell carcinoma
CASP3: caspase 3
CDKN1A: cyclin-dependent kinase inhibitor 1A, p21
CDKN1B: cyclin-dependent kinase inhibitor 1B, p27
EPHB2: ephrin type B receptor 2
PARP: poly ADP ribose polymerase
PC3: prostate cancer cell line
PI3K: phosphoinositide 3-kinase
PI: propidium iodide
SW-48: SW human colon adenocarcinoma
TP53: tumor suppressor p53
TZD: thiazolidinediones
