# Supplementary figures and images for: Peroxisome Proliferator Activated Receptor Gamma Sensitizes Non-small Cell Lung Carcinoma to Gamma Irradiation Induced Apoptosis
Source: Front Genet. 2019 Jun 13;10:554. doi: 10.3389/fgene.2019.00554 (PMC6585470; doi:10.3389/fgene.2019.00554)

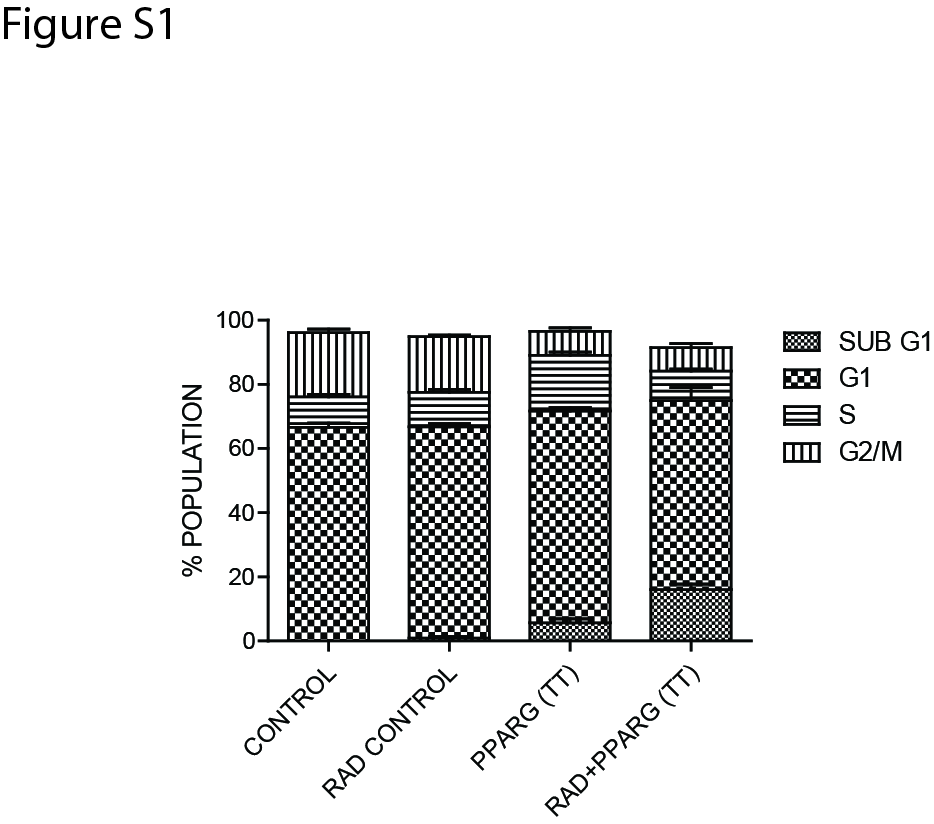

Supplement: FIGURE S1 — PPARG treatment led to accumulation of cells in S phase which on treatment with PPARG+radiation move toward cell death. Various phases of cell cycle after different treatment has been depicted. A549 cells were transfected with PPARG (PPARG (TT) and irradiated 24 h post-transfection, which is 48 h after transfection. These samples which included CONTROL, RAD CONTROL (5 Gy), PPARG (TT), and RAD+PPARG (TT) were processed for cell cycle analysis. The error bar represents standard deviation in which ∗P < 0.05, ∗∗P < 0.01, and ∗∗∗P < 0.001. The experiment was done in triplicate (n = 3). [file Image_1.TIF]
